# Multiple-point magnetic resonance acoustic radiation force imaging

**DOI:** 10.1002/mrm.27477

**Published:** 2018-09-26

**Authors:** Henrik Odéen, Joshua de Bever, Lorne W. Hofstetter, Dennis L. Parker

**Affiliations:** 1 Department of Radiology and Imaging Sciences, University of Utah, Salt Lake City, Utah; 2 Department of Radiology, Stanford University, Palo Alto, California

**Keywords:** acoustic radiation force imaging, ARFI, FUS, HIFU

## Abstract

**Purpose:**

To implement and evaluate an efficient multiple-point MR acoustic radiation force imaging pulse sequence that can volumetrically measure tissue displacement and evaluate tissue stiffness using focused ultrasound (FUS) radiation force.

**Methods:**

Bipolar motion-encoding gradients were added to a gradient-recalled echo segmented EPI pulse sequence with both 2D and 3D acquisition modes. Multiple FUS-ON images (FUS power > 0 W) were interleaved with a single FUS-OFF image (FUS power = 0 W) on the TR level, enabling simultaneous measurements of volumetric tissue displacement (by complex subtraction of the FUS-OFF image from the FUS-ON images) and proton resonance frequency shift MR thermometry (from the OFF image). Efficiency improvements included partial Fourier acquisition, parallel imaging, and encoding up to 4 different displacement positions into a single image. Experiments were performed in homogenous and dual-stiffness phantoms, and in ex vivo porcine brain.

**Results:**

In phantoms, 16-point multiple-point magnetic resonance acoustic radiation force imaging maps could be acquired in 5 s to 10 s for a 2D slice, and 60 s for a 3D volume, using parallel imaging and encoding 2 displacement positions/image. In ex vivo porcine brain, 16-point multiple-point magnetic resonance acoustic radiation force imaging maps could be acquired in 20 s for a 3D volume, using partial Fourier and parallel imaging and encoding 4 displacement positions/image. In 1 experiment it was observed that tissue displacement in ex vivo brain decreased by approximately 22% following FUS ablation.

**Conclusion:**

With the described efficiency improvements it is possible to acquire volumetric multiple-point magnetic resonance acoustic radiation force imaging maps, with simultaneous proton resonance frequency shift MR thermometry maps, in clinically acceptable times.

## 1 | INTRODUCTION

Focused ultrasound (FUS) is a totally noninvasive interventional technique in which large aperture ultrasound transducers are used to focus ultrasound energy into the body. The large aperture diffuses the energy entering the body over a large area, resulting in minimal tissue heating in the ultrasound near-field, and results in a converging high-intensity focal spot at the intended treatment location. Focused ultrasound has been used to treat a wide variety of benign and malignant tumors in various locations,^1–3^ bone metastasis,^4,5^ and neurological pathologies such as movement disorders and epilepsy.^6,7^

One challenge with ablative FUS treatments is to accurately determine the treatment endpoint. Currently, the treatment endpoint is estimated from the thermal dose in terms of cumulative equivalent minutes at 43°C (CEM43),^8^ which is a nonlinear function of temperature and time. Generally, tissue is considered ablated when it reaches a thermal dose of 240 CEM43. However, studies in rabbit and porcine muscle and brain have shown that lesions can be created at substantially lower doses in the range of 5 to 20 CEM43.^9–11^ Despite this, clinical treatments often exceed 240 CEM43 by a large margin due to the difficulty in controlling the delivery of thermal dose and the desire to increase the certainty of cell death. For tissues in which lesions can be created at lower thermal doses, this unnecessarily prolongs treatments, reducing treatment efficiency, and elevates the risk of damaging healthy tissue.

Accurate determination of thermal dose requires tissue temperature to be measured accurately over time. Magnetic resonance imaging can accurately measure temperature change in aqueous tissue using the proton resonance frequency shift (PRFS) method,^12^ but temperature measurements in adipose tissue are still not clinically available. In addition, because the PRFS method measures only temperature change, accurate absolute temperatures are obtained only if the initial temperature is known or can be estimated accurately. Because the dose rate doubles for every degree centigrade increase in temperature, small errors in temperature result in large errors in the calculated thermal dose. Because of uncertainty in temperature measurements and variations in tissue microstructure and perfusion, lesions have been observed to be created over a wide range of thermal dose values.^9–11^

Treatment endpoint can also be assessed using the non-perfused tissue volume measured with contrast-enhanced (CE) MRI. However, if the MR contrast agent is administered before FUS ablation, it can be temporarily trapped in tissue for up to 2 hours^13^ and can induce severe susceptibility artifacts, resulting in errors in subsequent PRFS measurements. For this reason, dynamic interrogations throughout the treatment are not advisable, and CE MRI should only be performed after treatment.^13,14^

Because of the uncertainty in lesion creation with thermal dose^9–11^ and the problems associated with dynamically applying CE MRI during the ablation process, several investigators have suggested using changes in tissue mechanical properties as an alternative or complement to thermal dose and CE MRI. Studies on imaging tissue elastography using both ultrasound shear wave imaging (SWI) and MR elastography (MRE) have shown that tissue stiffness changes irreversibly even at low levels of thermal dose.^15–17^ Diagnostic ultrasound systems can image tissue displacement and shear waves resulting from the acoustic radiation force impulses (ARFI) caused by short (~10–100 µs), intense FUS pulses,^18–20^ and this has been used to detect changes in shear wave velocity after tissue ablation.^16,21–26^ Magnetic resonance elastography uses an external driver to induce shear waves in the tissue while imaging tissue motions with phase-contrast MRI.^27,28^ Magnetic resonance elastography has been used to evaluate changes in shear wave velocity from thermal ablations such as FUS and laser.^15,29,30^ In ex vivo bovine tissue, the elastic shear wave modulus was found to increase close to linearly with the deposited FUS energy.^15,29^ Although both ultrasound ARFI and MRE are promising approaches for detecting tissue elasticity changes, they face logistical challenges when applied to endpoint evaluation for FUS treatments. The use of ultrasound imaging would require a dedicated imaging transducer within the FUS system,^23^ and although this has been done for other purposes, no such system is yet commercially available. Combining MRE and FUS is also challenging, and although simultaneous PRFS and MRE have been described,^31^ the system is substantially more complex than a standard FUS system.

It would therefore be beneficial if the tissue mechanical properties could be interrogated by the FUS system itself. The tissue displacement caused by the radiation force from a FUS sonication can be monitored and measured using MR-ARFI. By synchronizing FUS impulses with motion-encoding gradients (MEGs), the relatively small tissue displacement, on the order of tens of micrometers, can be encoded into the phase of the MR image. Different approaches for the MEGs, such as monopolar and bipolar, and different pulse sequences, such as gradient-recalled echo (GRE) and spin echo, have been described.^32–39^ The tissue displacement caused by the radiation force can be used to noninvasively measure relative tissue stiffness,^40^ locate the focal spot,^17,39^ or correct phase aberrations caused by aberrating media such as the skull or ribs.^36,38,41–45^ Methods to simultaneously measure tissue displacement with MR-ARFI and tissue temperature change using the PRFS method have further been described.^34,36,46,47^

Although MR-ARFI has been used previously to interrogate changes in tissue stiffness, these measurements have only been performed for 1 small focal region. To use tissue stiffness changes to determine the treatment endpoint, it will likely be necessary to interrogate a larger volume of tissue, which will be far too time-consuming to perform with existing MR-ARFI techniques.

In this work we present a multiple-point MR-ARFI (mpARFI) technique that is capable of efficiently acquiring volumetric displacement maps covering a large volume. Bipolar MEGs were implemented in a GRE-segmented (i.e., multishot) EPI pulse sequence, capable of performing both 2D and 3D imaging. Acquisition time was reduced by interleaving multiple image volumes in which the FUS was electronically steered to several spatial positions with a single reference image without FUS. Further efficiency gains were realized through partial Fourier acquisitions, k-space subsampling with parallel imaging, and sonicating the FUS in multiple spatial locations both during a single TR and during a single MEG lobe. Because the mpARFI was implemented in a GRE sequence, the reference image acquired without FUS can further be used to calculate accurate PRFS temperature maps. The technique is demonstrated for both 2D and 3D imaging, and experiments are performed in tissue-mimicking phantoms and ex vivo porcine brain. In the porcine brain it is shown that the measured displacement decreases after ablation due to increased tissue stiffness. The described method can potentially be useful both as a treatment evaluation tool to probe stiffness of ablated tissue, and as a diagnostic tool to efficiently investigate differences in mechanical properties between, for example, malignant and healthy tissue.^48,49^

## 2 | METHODS

### 2.1 | Pulse sequence

Bipolar MEGs were implemented in a GRE-segmented (i.e., multishot) EPI pulse sequence with the option to do monopolar frequency encoding (i.e., flyback readout). Before and after the EPI readout train, 2 FID field navigator echoes were acquired to monitor the B_0_ field evolution during the acquisition.^50^ The navigator echoes can be used to correct the phase of each k-space line to compensate for B_0_ drift due to respiration or gradient heating from using large MEGs. To provide flexibility, the sequence is implemented to enable both 2D and 3D acquisitions, to allow either fast single-slice displacement imaging or volumetric displacement maps.

**FIGURE 1:**
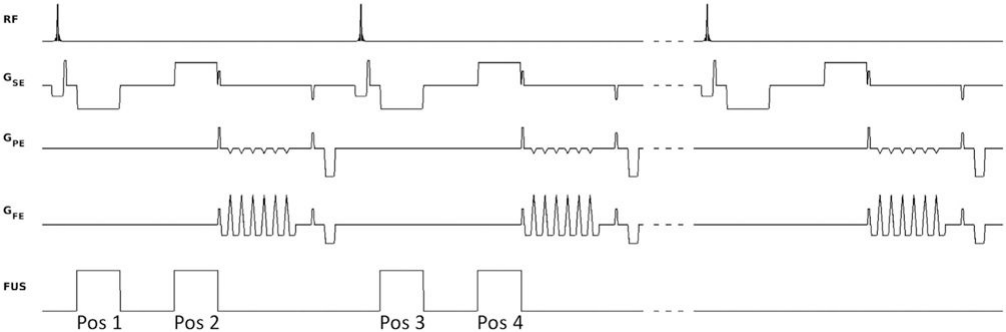
Pulse sequence diagram. Shown are 2 TRs with FUS ON (FUS power > 0 W), interleaved on the TR level with 1 TR with FUS OFF (FUS power = 0 W), in this case encoding 2 displacement positions per TR (one on each MEG lobe). The FUS is synchronized with the motion-encoding gradients (MEGs) using optical triggers. By electronically steering the FUS to different spatial positions for the positive and negative MEG lobe in a single TR, 2 different acoustic radiation force impulses (ARFI) displacement positions (labeled “Pos 1” and “Pos 2”) can be encoded into a single image. By electronically steering to 2 different positions during each of the 2 MEG lobes (not shown), a total of 4 different positions can be encoded into a single image

The ultrasound pulses were synchronized with the MRI acquisitions using optical triggers. In its simplest form, mpARFI sonicates a single spatial position per TR, and the FUS is triggered at the start of the second MEG lobe (Figure 1). Multiple 2D or 3D image volumes with the FUS-ON (i.e., Power [P] > 0 W) and a single image volume with the FUS-OFF (i.e., P = 0 W) are interleaved on the TR level.^51^ All FUS-ON images use electronic steering to move the FUS focal spot to a separate spatial position. The FUS-ON images accrue phase from both tissue displacement during the MEG, Δ*ϕ*_*D*_, and from tissue heating, Δ*ϕ*_*T*_, as Δ*ϕ*_*ON*_ = Δ*ϕ*_*D*_ + Δ*ϕ*_*T*_ according to

(1) $$\Delta \phi_{D}=\gamma \int_{0}^{\tau }MEG(t)\;\Delta D(t)\;dt$$

(2) $$\Delta \phi_{T}=\gamma \alpha B_{0}TE\Delta T$$

where *γ* is the gyromagnetic ratio (in Hz/T), *MEG* (*t*) are the time-dependent MEGs (in mT/m), Δ*D* is the tissue displacement (in m), τ is the duration of the MEG (in seconds), *α* is the PRFS constant (assumed to be 0.010 ppm/°C throughout this work), B_0_ is the main magnetic field strength (in T), and Δ*T* is the temperature change (in °C). The FUS-OFF image will only accrue phase due to tissue heating, Δ*ϕ*_*T*_. Because of the TR-level interleaved acquisition, the phase accrued due to temperature is identical for the FUS-ON and FUS-OFF images. By performing a complex subtraction between the FUS-OFF image and all FUS-ON images, phase-difference images with phase that is proportional to the induced tissue displacement can be achieved. Using Eq. (1), effective displacement maps can be computed from the phase-difference images. The FUS-OFF image can further be used to calculate an accurate PRFS temperature map, as it was interleaved with the FUS-ON images.^51^ This can be done either by acquiring a baseline image before the start of the mpARFI experiment, or by using referenceless PRFS reconstruction.^52,53^

**FIGURE 2:**
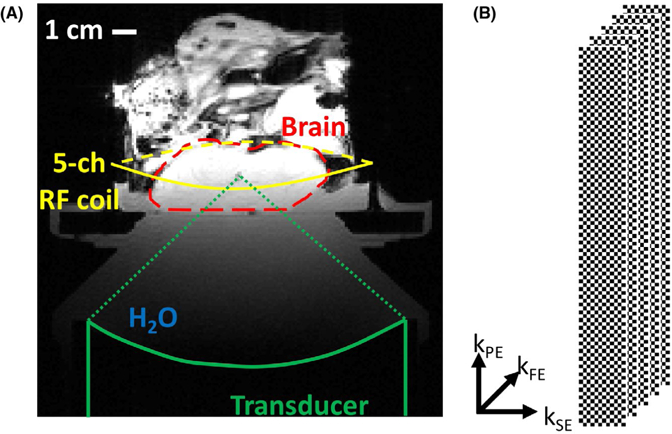
Experimental setup and k‐space sampling scheme. A, Experimental setup with FUS transducer coupled to phantom and ex vivo brain with bath of degassed and deionized water. A 5‐channel RF receive‐only coil was positioned around the sample. The approximate extent of the porcine brain within the skull with craniectomy is outlined in the dashed red line. B, The CAIPIRINHA sampling pattern used for 3D parallel imaging. For R = 2, the phase‐encoding slice‐encoding plane is subsampled with a “checker board” pattern, created by the shift of Δ = 1 from 1 k‐space slice encoding (k_SE_) to the next. This effectively shifts any remaining aliasing artifacts after reconstruction toward the corners of the 3D image volume

**FIGURE 3:**
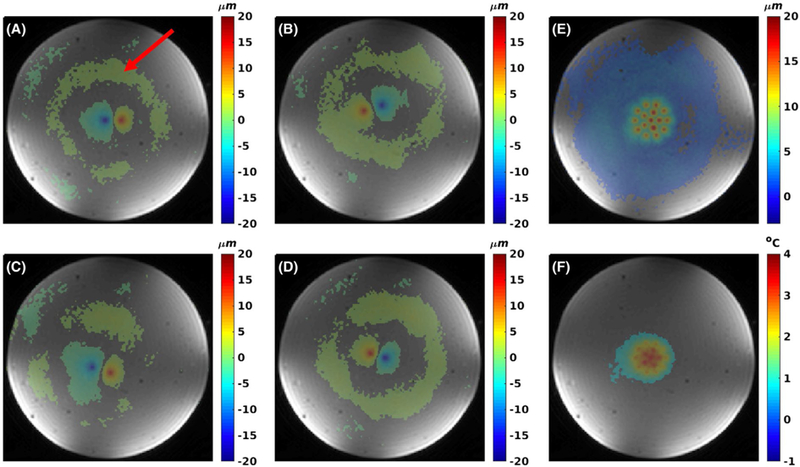
Two sonications per TR case of multiple‐point ARFI (mpARFI). A‐D, Individual points encoded using 2 sonications per TR for 3D acquisition. E, The corresponding maximum intensity projection (MIP) of all 16 points. The negative point is encoded first, and the created shear wave can be seen as a “ring” of positive displacement (red arrow in [A]) as encoded by the second MEG lobe. F, Proton resonance frequency shift (PRFS) temperature map from the FUS‐OFF image

Three different approaches were investigated to improve the efficiency of acquiring the mpARFI data. First, for 3D data, partial Fourier encoding in the slice-encoding direction was investigated by retrospectively using only part of the acquired k-space data. The partial Fourier data were reconstructed using an iterative phase–preserving projection onto convex sets (POCS) algorithm.^54^ Second, parallel imaging using GRAPPA^55^ for 2D imaging and CAIPIRINHA^56^ for 3D imaging was investigated. For 2D imaging, the k-y phase-encoding direction was evenly subsampled by sampling every Rth phase-encoding line. For 3D imaging, every Rth phase-encoding line was sampled in slice 1, and the sampling pattern was then shifted by Δ for the following slice, and so on (Figure 2). Throughout this work, R = 2 was used for both 2D and 3D imaging, and Δ = 1 was used for 3D imaging. Third, multiple displacement positions were encoded into a single image by sonicating at more than one location per TR, in which each sonication is electronically steered to a new spatial position (Figure 3). In this work we investigated performing both 2 and 4 sonications per TR. For 2 sonications per TR, one sonication is performed on each MEG lobe. Because the polarity of the 2 MEG lobes is different, 1 position will get encoded with a positive phase change and the other position with a negative phase change. For 4 sonications per TR, the FUS is steered to 2 different spatial locations during each of the 2 MEG lobes.

**FIGURE 4:**
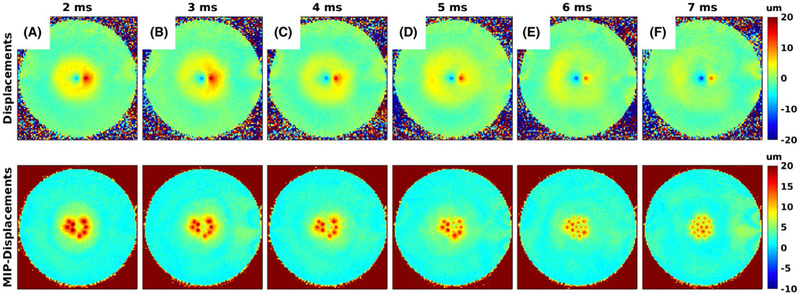
Effect of spacing between MEGs. A‐F, Effect of increasing the spacing between the MEGs from 2 ms to 7 ms for the case with 2 sonications per TR. The top row shows 1 of the individual displacement maps, and the bottom row shows the corresponding MIP of 8 individual displacement maps. In the top row the first encoded position shows up as negative, and the second encoded position shows up as positive, as the MEG polarity is switched. The shear wave from the first position gets encoded during the second MEG lobe and is therefore also positive. For small pause times the second position coincides spatially with the shear wave, resulting in overestimated displacements. As the pause time is increased, the shear wave moves past the position of the second sonication, and accurate measurements are achieved

When multiple sonications per TR are performed, a pause or dead time is inserted between the 2 MEG lobes for 2 reasons: (1) If the tissue displaced during the first MEG lobe is still relaxing back to equilibrium when the second MEG lobe is applied, some of the accrued phase for the first position will get unwound due to the reversed MEG polarity (adding a pause between the MEG lobes ensures that the tissue in the first position is relaxed back to equilibrium before the second MEG lobe is applied); and (2) the displacement at the second sonication position can be affected by an expanding shear wave from the first sonication position. If displacement at the second position due to the shear wave from the first position is encoded during the second MEG lobe, the added phase change will result in overestimation of the actual displacement caused by the second sonication (Figure 4). Adding a pause between the 2 MEG lobes ensures complete tissue relaxation of the first sonication position and shear-wave propagation past the second position so that these effects are minimized. If the shear-wave speed in the tissue/material is known, the needed pause between the MEGs can be calculated. In this work we empirically found the necessary pause by performing fast 2D imaging and gradually increasing the pause until the effects of (1) and (2) were not observed (Figure 4).

### 2.2 | Experiments

All experiments were performed on a 3T MRI scanner (Prisma^FIT^, Siemens Medical Solutions, Erlangen, Germany) with imaging parameters summarized in Table 1. An in-house-built 5-channel RF receive-only coil, placed around the sample, was used for signal detection. The coil consisted of an inductively decoupled 4-channel phased array with an additional integrated single-loop coil. The FUS sonications were performed using an MR-compatible phased-array ultrasound transducer (256 elements, 950-MHz frequency, 13-cm radius of curvature, focal spot size full width at half maximum 2 × 2 × 8 mm, Imasonic, Voray-sur-l’Ognon, France), with accompanying hardware and software for mechanical positioning and electronic beam steering (Image Guided Therapy, Pessac, France), and in-house-developed hardware and software to synchronize the MR pulse sequence with the FUS sonications using fiber optic triggers. The transducer was coupled to the targets with a bath of de-ionized and degassed water (Figure 2A).

Experiments were performed in tissue-mimicking phantoms and ex vivo porcine brain. Three different tissue-mimicking gelatin phantoms (Ballistic Gelatin, Vyse Gelatin, Schiller Park, IL) were constructed in acrylic cylinders (10-cm inner diameter, 15-cm height) with varying bloom values (higher bloom value results in stiffness phantom material).^57^ Phantom 1 was a homogenous phantom made of 125-bloom gelatin. Phantom 2 was a dual-stiffness phantom with one half filled with 125-bloom gelatin and the other filled with 175-bloom gelatin. The halves were oriented such that the ARFI points were positioned in one or the other half. Phantom 3 was a dual-stiffness phantom in which the halves were filled with 125-bloom and 250-bloom gelatin. The phantoms were doped with Copper Sulfate (CuSO_4_) to improve imaging by shortening T_1_. The 125-bloom gelatin was doped with 2 mMol/L, and the 175-bloom and 250-bloom gelatins were doped with 5 mMol/L. Doping the gelatins differently made it easier to detect the edge between the 2 different gelatins in the magnitude images of the dual-stiffness phantoms. The 3 different bloom values (125, 175, and 250) have previously been shown to have Young’s modulus values of 9.5 ± 1.8, 18.8 ± 2.7, and 29.4 ± 4.7 kPa, respectively.^57^ For the ex vivo porcine brain experiments, a fresh, previously not frozen pig’s head was sourced from a local butcher. The skull cap was removed to allow the FUS access to the brain without having to pass through the skull bone. After the skull cap was removed, the remaining head was put in saline and degassed for approximately 30 minutes. The setup for the ex vivo brain experiment is shown in Figure 2A.

In the homogenous phantom, comparisons between 2D (experiments 1 and 2 in Table 1) and 3D (experiments 3 and 4 in Table 1) imaging, with 1 and 2 sonications per TR and parallel imaging reconstruction, was investigated. A separate experiment using the 2 dual-stiffness phantoms with the parameters from experiment 3 in Table 1 was performed to investigate whether the mpARFI technique is sensitive enough to detect stiffness differences between the different bloom-value phantoms. In all phantom experiments, 2 different displacement patterns were used: either a 4 × 4 grid of points (acquired by sampling 16 FUS-ON images interleaved with 1 FUS-OFF image) or 16 points in 2 concentric circles (the outer circle containing 10 points, the inner circle containing 5 points, and 1 point at geometric focus, for a total of 16 FUS-ON points, which were acquired interleaved with 1 FUS-OFF point). In the ex vivo brain, 4 sonications per TR were used (experiment 5 in Table 1) and further efficiency improvements were achieved by using partial Fourier imaging, parallel imaging, and combination of both partial Fourier and parallel imaging. Finally, mpARFI was acquired before and after tissue ablation (experiment 6 in Table 1) to investigate whether tissue-stiffness changes with ablation could be detected. For the ablation experiment, only 1 sonication per TR and no further scan-time reduction was applied. During the ablation sonication, the PRF temperatures were monitored with a 3D segmented EPI sequence without MEG. Before each experiment a T_1_ map was acquired to estimate the Ernst angle (i.e., optimal excitation flip angle) for each TR according to $\alpha_{E}=arccos\left(e^{-(TR/T_{1})}\right)$. All data were zero-filled interpolated (in-plane only for 2D data and in all 3 dimensions for 3D data) to 0.5-mm isotropic resolution to minimize partial volume effects.

### 2.3 | Data reconstruction

All data were reconstructed in MATLAB (R2017a, the MathWorks, Natick, MA). Displacement maps were calculated by performing complex subtraction between the FUS-OFF image and all FUS-ON images, and then scaling by the MEG duration and amplitude. The PRFS data were reconstructed from the sequence of FUS-OFF images assuming a PRF coefficient α = –0.010 ppm/°C and single baseline subtraction followed by referenceless reconstruction using a second-order polynomial fit to the unheated background phase.^52^ The baseline was acquired before the start of the experiment. The 2 FID field-navigator echoes were investigated and showed that field drift was not an issue in the current study, so no further correction using the echoes was necessary. Multicoil MR data were optimally combined using Roemer’s equation (i.e., Eq. 24 in Roemer et al., with further details given in Parker et al.).^58,59^ The partial-Fourier data were reconstructed using an iterative phase–preserving POCS algorithm.^54^ Displacement-to-noise ratio (DNR) values (Table 1) were calculated as the mean of the maximum displacements in each individual displacement position, divided by the spatial SD of a region of interest in pre-FUS baseline images.

## 3 | RESULTS

Examples of 4 individual displacement images for the 2 sonications-per-TR case are shown in Figure 3A–D. The 2 displacement positions in each image are encoded as positive and negative displacements due to the bipolar MEG gradients. Figure 3E shows a displacement maximum intensity projection (MIP), and Figure 3F shows the thermometry map, which is acquired interleaved with the displacement map, demonstrating that mpARFI can be acquired without causing significant tissue heating.

The results of adjusting the delay between lobes for 2 sonications per TR are shown in Figure 4. The ring in each image is the shear wave propagating away from the ARFI point on the first MEG lobe that is encoded by the second MEG lobe. For this phantom, this ring has passed the second point with a 7-ms delay between lobes.

**FIGURE 5:**
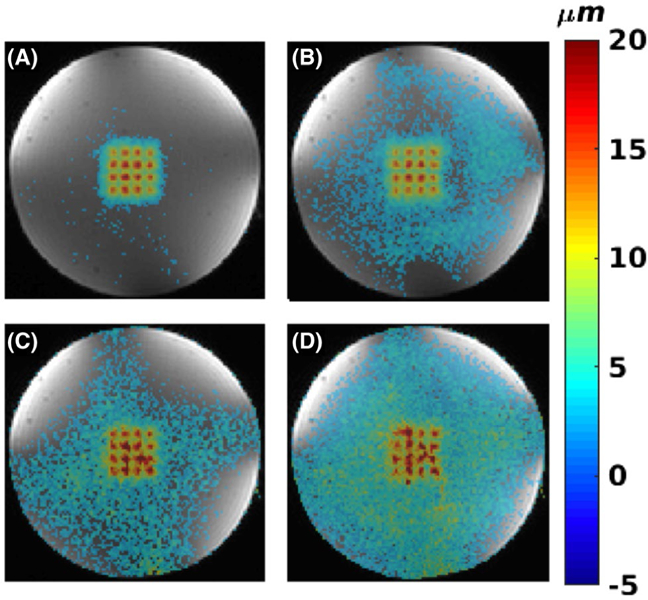
Homogenous phantom with 2D imaging. A,B, 1 and 2 sonications per TR, respectively. C,D, Parallel imaging with R = 2 for 1 and 2 sonications per TR, respectively

**FIGURE 6:**
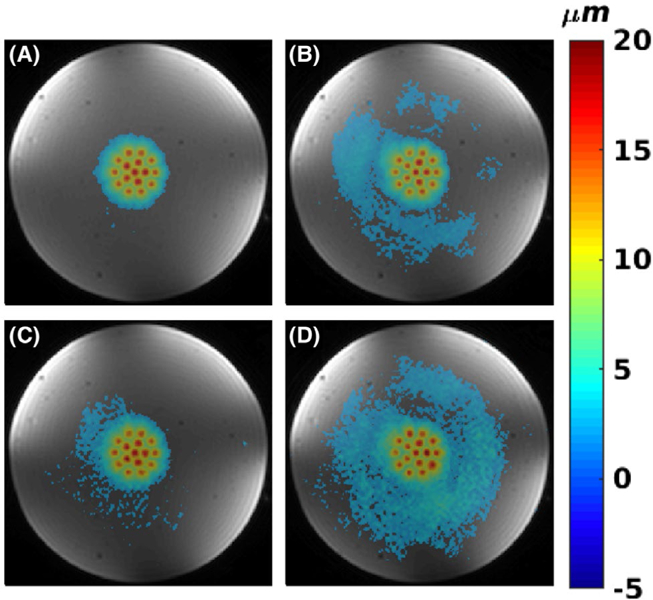
Homogenous phantom with 3D imaging. A,B, 1 and 2 sonications per TR, respectively. C,D, Parallel imaging with R = 2, for 1 and 2 sonications per TR, respectively

Figure 5 shows MIP displacement maps for 2D data in the homogenous phantom. Shown are the cases of doing 1 and 2 sonications per TR and doing 1 and 2 sonications per TR with parallel imaging applied. Doing only 2 sonications per TR or 1 sonication per TR with parallel imaging reduces the total scan time by a factor of approximately 2 (from 20.4 seconds to 10.8 seconds and 10.2 seconds, respectively). Performing both 2 sonications per TR and parallel imaging reduces the total scan time by a factor of approximately 4 (from 20.4 seconds to 5.4 seconds). It can be seen that performing 1 or 2 sonications per TR creates coherent displacement maps. Performing parallel imaging causes at least an $\sqrt{R}$ decrease in SNR, resulting in substantially noisier and noncoherent displacement maps. Figure 6 shows MIP displacement maps for 3D mpARFI data obtained with the concentric circle point arrangement and with 1 and 2 sonications per TR, without and with parallel imaging applied (resulting in scan times of 245 seconds, 130 seconds, 122 seconds, and 65 seconds, respectively). In the case of 3D imaging, the inherently higher SNR can be seen to produce coherent displacement maps for all 4 cases, even when both 2 sonications per TR and parallel imaging are used for a total scan time reduction of approximately 4 times.

**FIGURE 7:**
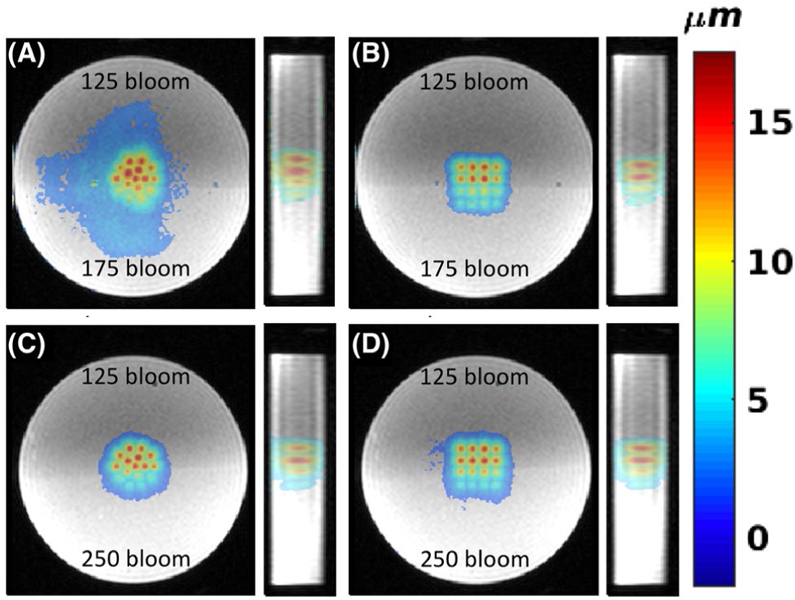
Three-dimensional mpARFI in dual-stiffness phantoms. A,B, Coronal and sagittal views of circular and 4 × 4 mpARFI trajectories in a dual-stiffness 125/175-bloom phantom, respectively. C,D Corresponding trajectories in a dual-stiffness 125/250-bloom phantom. To achieve the best contrast between the 2 different gelatins (which had different CuSO_4_ doping), the magnitude images shown are acquired with a 3D gradient-recalled-echo (GRE) sequence, whereas the overlaid displacement maps are acquired with the segmented EPI sequence

In Figure 7 the results for the 2 dual stiffness phantoms (125 of 175 bloom and 125 of 250 bloom, respectively) are shown. In both phantoms, substantially smaller displacements are seen in the lower half of the phantom, containing the stiffer 175 and 250 bloom gelatins. The higher CuSO_4_ concentration in the stiffer gelatin results in the higher MR signal in the underlying magnitude images.

**FIGURE 8:**
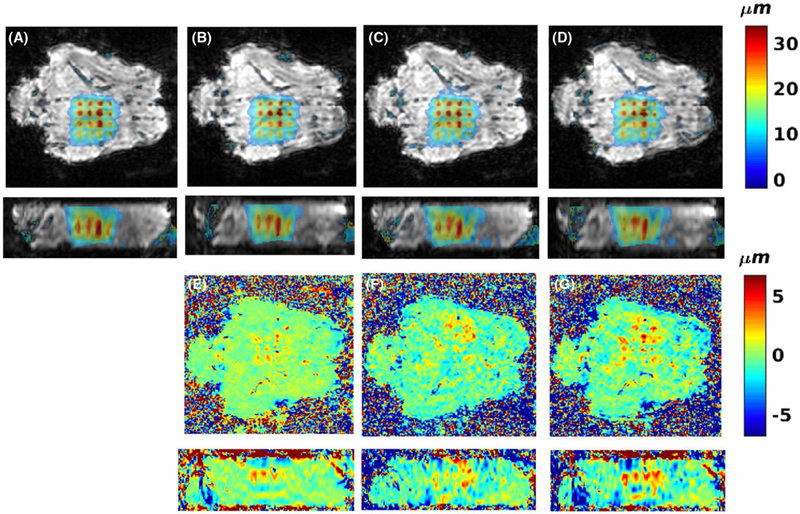
Effects of multiple sonications per TR, partial-Fourier, and parallel imaging on ex vivo mpARFI. Ex vivo porcine brain data using 4 sonications per TR for no additional speedup A, 33% speedup by partial Fourier (sampling 8 of 12 k-z slices) B, speedup of 50% by parallel imaging with R = 2 C, speedup of 67% by partial Fourier and parallel imaging D. E-G, Errors among (B), (C), (D) and (A), scaled to ±20% or the maximum measured displacement of 42.4 µm

**FIGURE 9:**
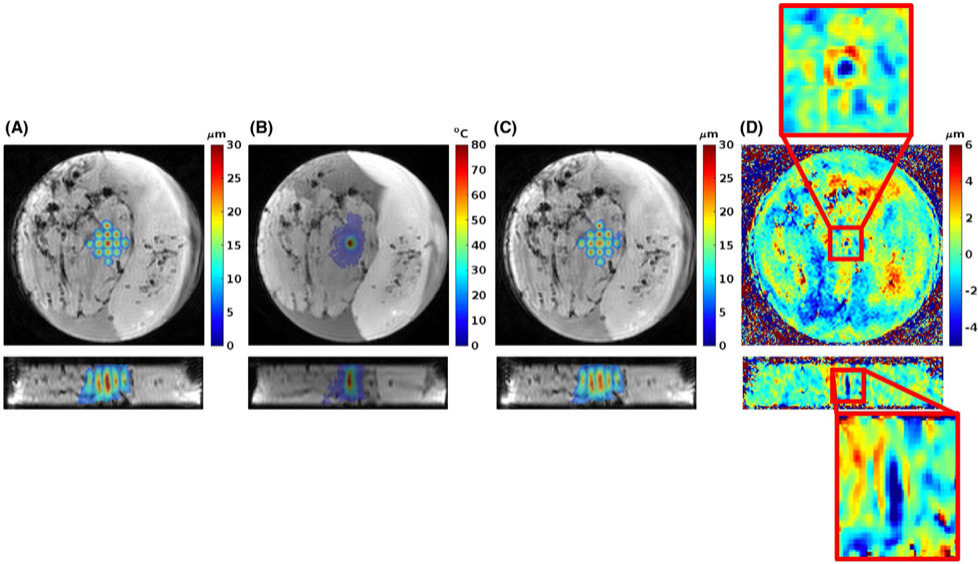
Multiple-point ARFI before and after tissue ablation. A, Two orthogonal views of MIP displacement map of 13 FUS-ON acquisitions before ablation, overlaid on magnitude image. B, Two orthogonal views of PRFS temperature rise after 200 W sonication for 40 seconds at geometric focus (data acquired with 3D GRE-segmented EPI sequence without MEG), overlaid on magnitude image. C, Two orthogonal views of MIP displacement map after ablation. D, Two orthogonal views of displacement difference between before and after ablation. Red boxes magnify difference around geometric focus where the ablation took place

Figures 8 and 9 show the results from the ex vivo porcine brain studies. All data in Figure 8 are acquired with 4 sonication per TR (i.e., 2 sonications/MEG lobe). Here, partial Fourier in the k-z slice-encoding direction is investigated by using only 8 of the 12 k-z encodes, resulting in a scan time reduction of 33%. Parallel imaging (CAIPIRINHA with R = 2 and Δ = 1), resulting in a scan-time reduction of 50%, and combination of parallel imaging (R = 2 and Δ = 1) with partial Fourier (8 of 12 k-z encodes) was also investigated. In all cases the error compared with no additional scan time reduction (i.e., just performing 4 triggers per TR) was below 20% of the maximum measured displacement of 42.4 µm. Figure 9 shows MIP displacement maps before and after a continuous sonication, 200 W for 40 seconds, resulting in a temperature rise of approximately 80 ºC. Also shown is the MIP displacement for the difference between pre-ablation and post-ablation, showing a decrease in displacement at the ablated region by approximately 22%.

As indicated by the DNR values in Table 1, the DNR decreases with the application of parallel imaging. The DNR is also lower for the multiple sonications-per-TR cases, as these use a slightly longer TE to accommodate for the spacing that is inserted between the MEG lobes. The slight increase in DNR for partial Fourier data (protocol 5, for ex vivo brain) is due to the application of a Hamming filter in k-space during the POCS reconstruction. This also results in a slight blurring of the focal spots, and hence the lower maximum displacement that can be seen in Table 1 for these cases. Comparing Table 1 and Figures 5, 6 and 8, it can qualitatively be seen that for DNR values greater than approximately 16, all individual displacement positions can be distinguished (Figure 5A,B, Figure 6A–D, and Figure 8A–D), whereas for lower DNR values in the range of 11 to 13 (Figure 5C,D), all focal spot positions are not readily distinguishable.

## 4 | DISCUSSION AND CONCLUSIONS

In this work we have described a mpARFI technique that can efficiently interrogate the stiffness of a 3D volume by interleaving multiple FUS-ON imaging volumes, each electronically steered to a different spatial location, with a single FUS-OFF image volume interleaved at the TR level. Using the mpARFI technique, scan times were reduced by up to 74% in phantoms (performing 2 sonications per TR and parallel imaging with R = 2) and up to 90% in ex vivo studies (performing 4 sonications per TR, parallel imaging with R = 2, and 2/3 partial Fourier). In the phantom, a single-slice 2D acquisition was performed in approximately 10 seconds and a 3D acquisition in approximately 60 seconds. In the ex vivo experiments, a 3D acquisition was acquired in approximately 20 seconds, making the total scan time feasible for the clinical setting. In addition to the mpARFI map, the technique simultaneously measures a PRFS temperature map. It was shown that the technique could accurately distinguish the interface between regions of a phantom with different stiffness, and in an ex vivo experiment detect changes in stiffness before and after FUS ablation.

The phantom experiments showed that the parallel imaging SNR penalty of at least $\sqrt{R}$, combined with the inherent $\sqrt{N_{z}}$ lower SNR of 2D versus 3D imaging (in which *Nz* is the number of slices in a 3D volume) resulted in poor quality displacement maps. It is, of course, possible to increase the number of signal averages, at the cost of increased scan time, for 2D imaging. For 3D imaging, coherent displacement maps could be reconstructed using a combination of 2 sonications per TR (reducing scan time by 47% by decreasing the total number of image volumes acquired from 16 FUS-ON + 1 FUS-OFF to 8 FUS-ON + 1 FUS-OFF) and parallel imaging with R = 2, for a total scan time reduction of 74%.

The experiments in dual-stiffness phantoms demonstrated that mpARFI was sufficiently sensitive to measure the expected difference in displacement between gelatins of 125, 175, and 250 bloom (which have previously been shown to have Young’s modulus values of 9.5 ± 1.8, 18.8 ± 2.7, and 29.4 ± 4.7 kPa, respectively).^57^ This is most clearly seen in Figure 7C–D, where the interface between the two phantoms is not aligned with the middle of the interrogated volume, but rather at a slight angle. For example, in Figure 7D, in the third row of points the 3 left-most points that are in the softer 125-bloom part of the phantom show greater displacement than the right-most point that is in the stiffer 250-bloom part of the phantom.

One challenge with the phantom study was that the gelatin supported shear waves better than ex vivo tissue. Figure 4 shows that a pause between MEG lobes of at least 7 ms was needed before the shear wave had propagated far enough, and lost enough amplitude, to not cause problems for the multisonication-per-TR cases. We attempted to do 4 sonications per TR in the phantoms, but the TE value necessary to solve the shear-wave problem was too long to give reasonable SNR. In the ex vivo data, shorter pauses could be used without causing interference between shear waves originating from an early point and displacement from later points. It can be hypothesized that the (ex vivo) brain tissue was more viscous than the phantom material, and therefore the shear wave amplitudes decreased more rapidly as has previously been observed in liver tissue.^60^ Further studies are necessary to validate this hypothesis.

Even though our limited ex vivo brain studies showed that the interfering shear waves during multiple sonications per TR were less of a problem ex vivo than in phantom, the shear wave velocity is tissue type–dependent and also affected by factors such as tissue temperature and disease state. For example, the shear wave velocity of (healthy) liver and pancreas has been shown to be in the range of 1.2 to 1.6 m/s, whereas kidney and spleen have values of 2.2 and 2.4 m/s, respectively.^61,62^ In terms of disease state, increased shear wave velocity in liver has been shown for fibrosis and cirrhosis, with values of 1.8 and 2.1 to 2.3 m/s, respectively.^61,63,64^ In terms of the temperature dependence of the shear wave velocity (and hence the directly proportional shear modulus), it has been showed that liver does not change stiffness until being heated to at least 45°C, whereas muscle-tissue stiffness varies in 4 successive steps between 25°C and 65°C, consistent with different levels of thermally induced protein denaturation.^65^ Tissue stiffness changes, both in vivo and ex vivo, have been shown to be closely linked to thermal dose, with, for example, a dose of 202 CEM43, resulting in an 8-fold stiffness increase in in vivo rat muscle, as measured by ultrasound SWI.^8,65,66^ Tissue stiffness in muscle is further directional-dependent due to the muscle fibers, and the transversal shear modulus has been shown to be approximately 1.80 times smaller than the longitudinal shear modulus.^65^ Because of all of these effects, careful validation of the mpARFI technique in each target and organ of interest will be necessary for in vivo applications, and challenges such as tissue boundaries (e.g., bone, bladder) will also need to be evaluated.

In the ex vivo studies using 4 sonications per TR (Figure 8 and protocol 5 in Table 1), rather long TR and TE were needed due to current hardware limitations on how closely spaced fiber optic triggers could be accepted by the FUS generator. The shortest combination of FUS pulse duration and spacing between FUS pulses that could be achieved was 7-ms-long pulses with 2.5-ms spacing between pulses. This resulted in MEG durations of 19 ms, and a pause between MEGs of 3 ms was found to work well in the ex vivo brain case. The long TR (73 ms) and large MEG area (caused by MEG amplitude of 50 mT/m and duration of 19 ms) resulted in significant T_2_* decay and diffusion effects, respectively, resulting in the rather low signal magnitude images seen in Figure 8. Despite the low signal, coherent displacement maps could be reconstructed from the data. It was shown in Figure 8 that scan time reductions of between 33% and 50%, using partial Fourier imaging or parallel imaging, could be achieved while keeping the error below approximately 10% to 15% of the maximum displacement. If both methods were combined, the error increased slightly to approximately 20% of the maximum displacement, but the scan time was reduced by 67% compared with just doing 4 sonications per TR. Doing 4 sonications per TR by itself sped up the acquisition by 71% (for the 16-point trajectory, instead of acquiring 16 FUS-ON + 1 FUS-OFF = 17 imaging volumes, only 4 FUS-ON + 1 FUS-OFF = 5 image volumes had to be acquired).

Figure 9 shows an initial study to investigate changes in tissue mechanical properties with thermal ablation. The same mpARFI protocol was run before and after a continuous 40-second sonication at 200 W, which created a temperature rise of approximately 80°C at the geometric focus. The decrease in displacement of approximately 22% after ablation agrees well with what has been reported previously in ex vivo muscle.^47^ For in vivo experiments, it has been found that rat brain became softer and rat leg muscle became stiffer with high thermal dose, indicating that different tissues may respond differently with thermal treatment.^66,67^ Although this agreement with the literature regarding ex vivo studies is encouraging, more ex vivo and especially in vivo experiments are needed to draw any real conclusions about how the tissue mechanical properties change with clinically relevant ablations. Even though just a single point was ablated in the current work, the described mpARFI technique can of course also be used to monitor “volumetric” ablations from multiple consecutively ablated focal points or ablation “trajectories.” Furthermore, it can be postulated that the mpARFI technique can be combined with high duty-cycle FUS to simultaneously ablate tissue and measure temperature and displacement in multiple focal positions, similar to what has previously been described for single-point ablations.^47,51^

Three different techniques for improving acquisition efficiency and decrease acquisition time were investigated in this work: (1) partial Fourier imaging, (2) parallel imaging, and (3) sonicating at multiple spatial locations in a single TR, which is the most novel aspect of this work and unique to the mpARFI technique.

Partial Fourier imaging is a standard approach to reduce scan time but can lead to errors in the reconstructed images. In the case of mpARFI, we showed that scan-time reductions of up to 33% could be achieved without introducing substantial errors in the displacement maps. Many partial Fourier reconstruction approaches produce only magnitude images, and do not provide the image phase required for ARFI and MR thermal imaging. In this work, a phase-preserving iterative POCS algorithm was used.^54^

Parallel imaging is another standard approach to reduce scan time, which relies on the difference in coil sensitivity between multiple RF-receive coils. In this work, a 5-channel coil was used for a scan-time reduction of R = 2. It has previously been shown that the k-space subsampling pattern used affects the parallel imaging reconstruction, and that by controlling the aliasing, as in CAIPIRINHA, improved imaging can be achieved. For the 3D imaging in this work, CAIPIRINHA with a Δ shift of 1 was applied. The Δ-shift results in aliasing not only in the subsampled phase-encoding direction, but because the sampling pattern shifts from slice to slice, it also aliases in the slice-encoding direction, effectively moving the aliasing artifact to the edges of the 3D imaging volume.

Encoding multiple displacement positions into a single image is specific to mpARFI. It was shown that substantial scan-time reductions can be achieved by encoding up to 4 displacements in a single image. In this work the mpARFI trajectories had 13 or 16 positions with FUS-ON, but the limit for number of points is really only set by how far the transducer can electronically steer without losing substantial power, and how quickly the driving hardware can switch between spatial positions. For 3D imaging it is also possible to do multiple “layers” of displacement maps in the slice-encoding direction. We note that the rapid steering between points used by mpARFI is only possible for phased array transducers. However, most of all clinically used transducers are steerable phased arrays.

As shown in Figure 4, the feasibility of doing multiple sonications per TR depends on the shear waves that are created and how they interact with the points that are encoded during the second MEG lobe. This was a much bigger challenge in the phantom study than in the ex vivo tissue studies. Current hardware limitations further limited the efficiency improvements that could be achieved in the ex vivo case with 4 sonications per TR. To the extent that pulse durations and intervals can be reduced, the MEG durations could be reduced and the interval between lobes chosen to minimize shear wave contributions to displacement. If 2 5-ms-long FUS pulses could be applied during each MEG, keeping the MEG duration to 10 ms and spacing the lobes by approximately 5 ms would reduce the TR by 16 ms down to 57 ms, and simultaneously improve SNR while reducing scan time from 60 seconds to 48 seconds. Combining this with parallel imaging and partial Fourier imaging, the total acquisition time could be reduced to 16 seconds.

The results presented in this work show that mpARFI could potentially be a useful tool for evaluating changes in tissue mechanical properties after FUS ablations. It was shown in phantoms that mpARFI can detect the interface between different stiffness gelatins, and in ex vivo porcine brain that mpARFI can detect decreased displacements after a FUS ablation. Multiple-point ARFI can potentially also be used as a diagnostic tool to investigate mechanical properties between, for example, suspected tumors and surrounding healthy tissue. The fact that the mpARFI sequence simultaneously measures displacement and temperature change is advantageous from a safety perspective.

## Figures and Tables

**TABLE 1:** Magnetic resonance and ultrasound parameters.

	TR, TE (ms)	FOV (mm)	Res (mm)	BW (Hz/px)	ETL	FA (deg)	t_acq_ (s)	MEG Amp., Dur., Pause (mT/m, ms, ms)	FUS Amp, Dur (W, ms)	ΔD (µm)	DNR
Phantom											
1. 2D 1 son./TR	100, 24	160 × 100 × 2.2	1.25 × 1.25 × 2.2	752	7	35	20.4 (R = 1) 10.2 (R = 2)	50, 5, 0	103, 5	17.1 ± 2.3 (13.0‐21.1) 21.1 ± 3.9 (17.2‐29.8)	25.5 12.5
2. 2D 2 son./TR	100, 32	160 × 100 × 2.2	1.25 × 1.25 × 2.2	752	7	35	10.8 (R = 1) 5.4 (R = 2)	50, 5, 7	103, 5	16.1 ± 2.0 (13.3‐19.9) 21.2 ± 4.0 (15.4‐31.1)	17.5 11.1
3. 3D 1 son./TR	100, 24	160 × 100 × 30	1.25 × 1.25 × 2.5	752	7	35	245 (R = 1) 122 (R = 2)	50, 5, 0	103, 5	16.2 ± 1.4 (14.8‐18.5) 16.1 ± 1.6 (14.4‐19.1)	37.9 21.8
4. 3D 2 son./TR	100, 32	160 × 100 × 30	1.25 × 1.25 × 2.5	752	7	35	130 (R = 1) 65 (R = 2)	50, 5, 7	103, 5	15.7 ± 1.4 (14.0‐18.4) 16.3 ± 1.4 (14.0‐19.7)	19.6 16.7
Ex vivo											
5. 3D 4 son./TR	73, 55	160 × 120 × 30	1.25 × 1.25 × 2.5	752	7	25	61 41 (PF) 31 (PI) 20 (PF + PI)	50, 19, 3	200, 7	29.3 ± 5.7 (22.0‐38.7) 27.3 ± 5.4 (18.4‐37.3) 28.9 ± 5.5 (21.8‐38.4) 26.9 ± 5.4 (19.0‐36.4)	29.8 33.3 19.4 22.0
6. 3D 1 son./TR	42, 12	160 × 110 × 30	1.25 × 1.25 × 2.5	752	7	20	91.7	50, 5, 0	200, 5		
7. MRTI ablation	26, 11	160 × 110 × 30	1.25 × 1.25 × 2.5	752	7	20	4.1	—	200, 40 s		

Acquisition times are reported for the full displacement maps, including the FUS‐OFF image. For experiments 1‐4, the acquisition times are given for no parallel imaging (R = 1) and for using parallel imaging (R = 2). For experiment 5, the acquisition times are given for partial Fourier (PF), parallel imaging (PI), and PF + PI. Abbreviations: Res, resolution; BW, frequency encoding bandwidth; ETL, echo train length; FA, flip angle; t_acq_, acquisition time; MEG Amp/Dur/Pause, MEG amplitude/duration/pause (i.e., spacing between MEG lobes); FUS Amp, Dur, FUS amplitude and duration; ΔD, mean of maximum displacements for each multipoint trajectory, ±SD of the points (the range of displacements is also listed); DNR, displacement‐to‐noise ratio calculated as the mean of the maximum displacements for all points, divided by the SD of the displacement measured in pre‐FUS baseline images.
